# Dark urine as the initial manifestation of COVID-19: a case report

**DOI:** 10.1186/s13256-021-03173-x

**Published:** 2021-12-02

**Authors:** Goar Egoryan, Sana Chaudry, Kritika Yadav, Tianyu Dong, Emre Ozcekirdek, Ece Ozen, Guillermo Rodriguez-Nava

**Affiliations:** 1 Department of Internal Medicine, AMITA Health Saint Francis Hospital, 355 Ridge Ave, Evanston, IL USA; 2 Department of Internal Medicine, AMITA Health Saint Joseph Hospital, Chicago, IL USA

**Keywords:** Rhabdomyolysis, Creatine kinase, COVID-19, Coronavirus, SARS-CoV-2

## Abstract

**Background:**

Rhabdomyolysis is defined as a syndrome consisting of muscle necrosis and the release of intracellular muscle components into the bloodstream. Although rhabdomyolysis has been previously reported as an initial presentation or late complication of COVID-19, the data on it is still limited, and currently, there is no single case of COVID-19 in the literature that describes creatine kinase levels of more than 30,000 IU/L.

**Case presentation:**

A 50-year-old African–American male presented to the hospital with decreased urine output, dark urine color, and constipation for the past couple of days. He was found to have acute kidney injury, liver injury, and creatinine kinase of 359,910 IU/L, for which aggressive intravenous fluid therapy was given. Infectious workup resulted in positive severe acute respiratory syndrome coronavirus 2 polymerase chain reaction. Two days after admission, the patient became symptomatic from a coronavirus disease 2019: he developed fever and hypoxia, and was placed on supplemental oxygen and started on a 10-day course of dexamethasone. The patient responded well to the treatment and supportive care for coronavirus disease 2019 and was successfully discharged.

**Conclusion:**

Clinicians should be cognizant of atypical coronavirus disease 2019 presentations. The spectrum of damage of coronavirus disease 2019 is still an evolving topic, and more research is required to reveal the exact mechanisms by which severe acute respiratory syndrome coronavirus 2 leads to rhabdomyolysis.

## Background

From its discovery in late December 2019, severe acute respiratory syndrome coronavirus 2 (SARS-CoV-2) has caused global public health emergencies and economic crises. The typical spectrum of coronavirus disease 2019 (COVID-19) clinical manifestations includes dry cough, dyspnea, fever, chills, myalgias, anosmia, and less frequently headache, nausea, vomiting, and diarrhea. However, several extrapulmonary and atypical manifestations exist, such as cerebrovascular accidents, venous thromboses, pulmonary embolism, myocardial infarction, myocarditis and pericarditis, acute kidney injury (AKI), liver injury, conjunctivitis, Guillain-Barre syndrome, and encephalitis [1]. Rhabdomyolysis is defined as a syndrome consisting of muscle necrosis and the release of intracellular muscle components into the bloodstream. CK levels are typically significantly elevated, and myalgias with myoglobinuria may be present. The clinical presentation varies from an asymptomatic elevation of serum muscle enzymes to severe life-threatening disease, with the main concern being AKI. Although rhabdomyolysis has been previously reported as an initial presentation or late complication of COVID-19, the data on it is still limited, and currently, there is no single case of COVID-19 in the literature that describes CK levels of more than 30,000 IU/L. We report a case of rhabdomyolysis as the main and initial presentation of COVID-19, with an immensely elevated level of CK of more than 350,000 IU/L.

## Case presentation

A 50-year-old African–American male presented to the hospital with complaints of decreased urine output, dark urine color, and constipation for the past couple of days. The patient denied any other symptoms. Medical history was remarkable for type 2 diabetes, hypertension, hyperlipidemia, and legal deafness and blindness. The patient denied smoking, use of any recreational drugs, or alcohol abuse. Home medications included atorvastatin 20 mg daily that the patient had been taking for the past 2 years, lisinopril 5 mg daily, and metformin 500 mg daily. Vital signs upon admission were grossly unremarkable. Initial laboratory studies revealed significant elevation of aspartate aminotransferase (AST) and alanine aminotransferase (ALT) (AST 2222 IU/L, ALT 432 IU/L), creatinine (Cr) of 2.29 mg/dL, hypocalcemia (ionized calcium of 0.92 mmol/L), hyponatremia (127 mmol/L), and a moderately increased high-sensitivity troponin level (HST) of 102 pg/mL. Further AKI workup yielded an enormously high level of CK 359,910 IU/L (no laboratory errors were reported, the result was consistent on repeated measurements) and hyperuricemia (11.4 mg/dL); urinalysis showed cloudy urine with a large amount of blood and only 3–5 red blood cells per high-power field.

Additional tests demonstrated a very high aldolase level (> 250 U/L, reference range 1.2–7.6 U/L) and lactate dehydrogenase (> 6000 IU/L, reference range 140–271 IU/L). Hence, the diagnosis of rhabdomyolysis was made. Since statin use alone did not seem enough to cause such high CK levels, further workup was conducted. Urine toxicology screen, serum levels of acetaminophen and salicylates were all negative. Immunoglobulin G (IgG) antibodies to 3-hydroxy-3-methylglutaryl-coenzyme A reductase did not yield a positive result. Infectious studies were negative for influenza virus, respiratory syncytial virus, HIV 1/2, Epstein–Barr virus, cytomegalovirus, herpes simplex virus, coxsackievirus, *Mycoplasma pneumoniae*, *Streptococcus pneumoniae*, and *Legionella pneumophila*; respiratory viral panel and blood cultures also came back negative. Since computed tomography of abdomen and pelvis done in the emergency department revealed bibasilar ground-glass opacities, COVID-19 PCR was also sent, which turned out to be positive, although initially, the patient did not have any symptoms of respiratory tract disease.

**Fig. 1:**
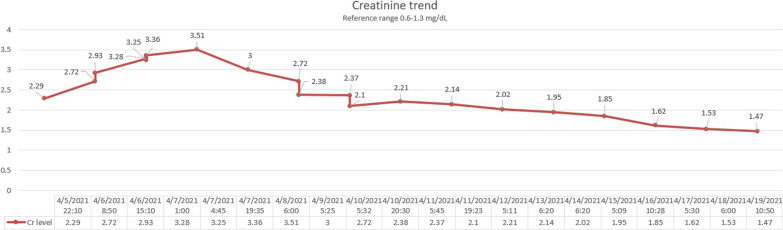
Trend of patient’s serum creatinine

**Fig. 2:**
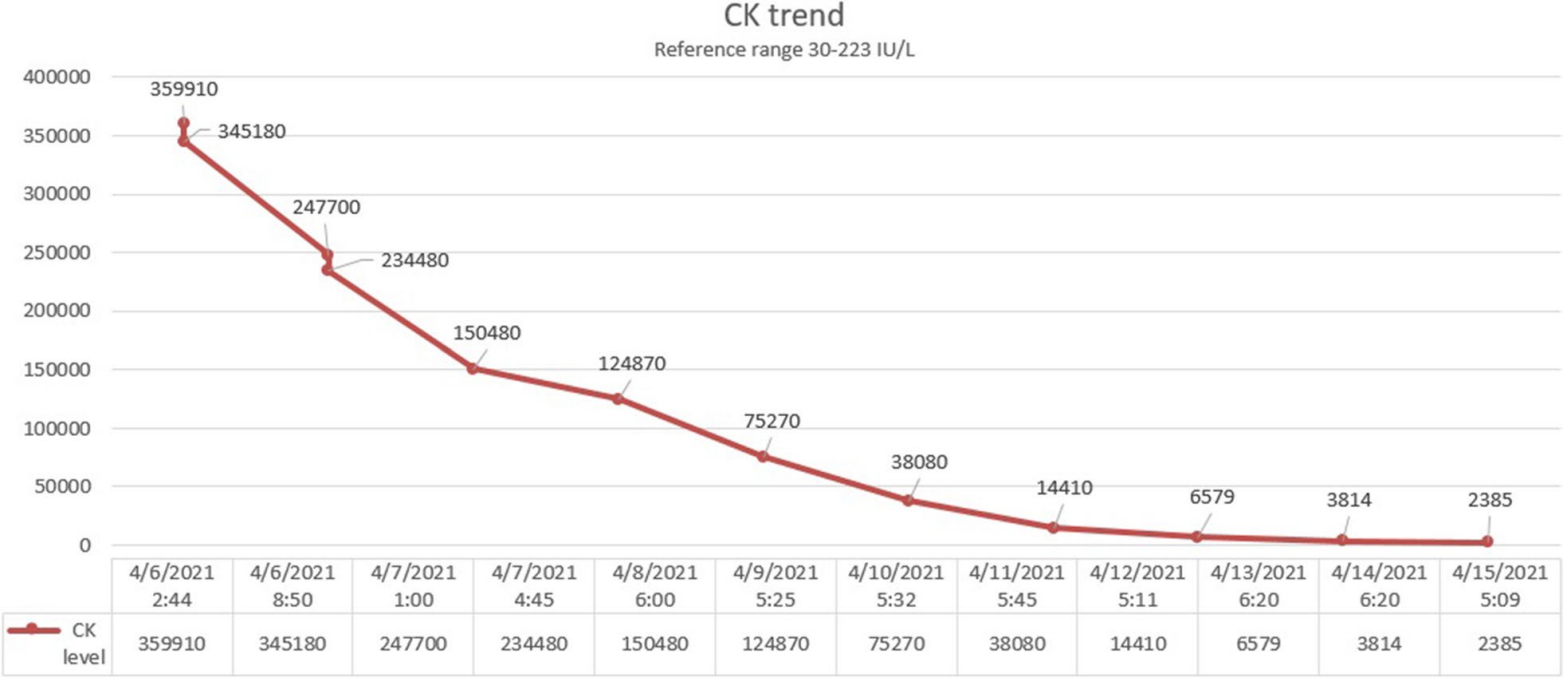
Trend of patient’s creatinine kinase

**Fig. 3:**
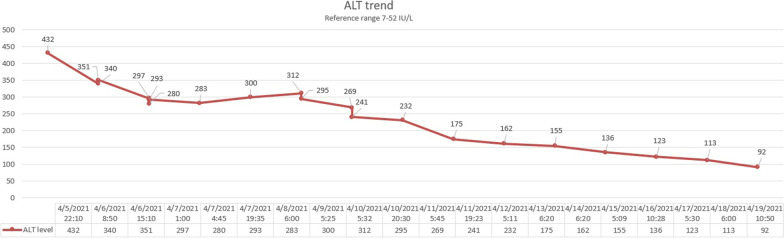
Trend of patient’s alanine aminotransferase

**Fig. 4:**
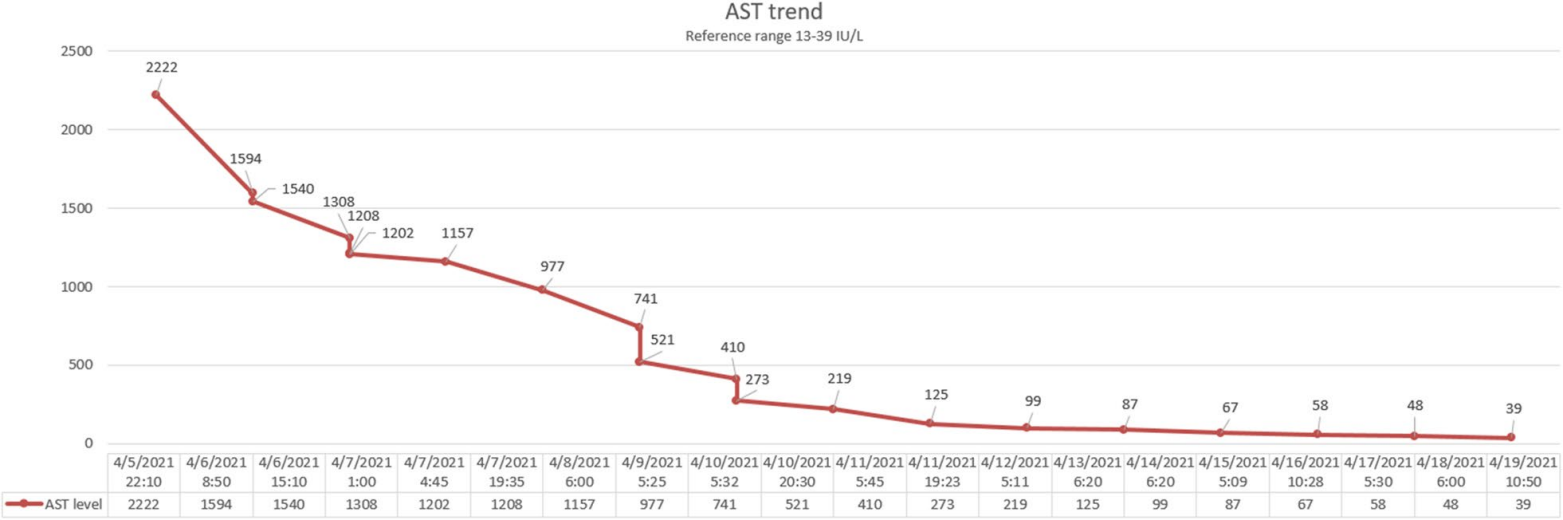
Trend of patient’s aspartate aminotransferase

The patient was started on aggressive intravenous fluids resuscitation. Eventually, his kidney function improved with a decrease in Cr (Fig. 1) and significant down trending of CK (Fig. 2). Besides that, the level of transaminases started to go down synchronically with the initiation of rhabdomyolysis treatment (Figs. 3, 4). Viral hepatitis panel was negative, gamma-glutamyl transferase (GGT), total bilirubin, and alkaline phosphatase levels remained normal throughout his hospitalization. The conclusion was made that isolated transaminitis resulted from rhabdomyolysis, and hepatotoxicity from COVID-19 is known. Two days into the hospitalization, the patient became symptomatic from a COVID-19 standpoint: he developed fever and hypoxia, and was placed on supplemental oxygen and started on a 10-day course of dexamethasone. Overall, the patient responded well to the treatment and supportive care for COVID-19, and was successfully discharged home.

## Discussion

Rhabdomyolysis is a syndrome caused by injury to skeletal muscle and involves leakage of large quantities of potentially toxic intracellular contents into the plasma, such as myoglobin. After muscle injury, massive plasma myoglobin levels exceed the protein binding of haptoglobin, precipitating in the glomerular filtrate, causing renal tubular obstruction, direct nephrotoxicity (ischemia and tubular injury), intrarenal vasoconstriction, and AKI [2–4]. The classic triad of rhabdomyolysis consists of myalgias, generalized weakness, and dark urine. However, in practice, presentations vary considerably. The known common causes of rhabdomyolysis are trauma and crush injuries, infections (of which the most frequently implicated agent is the influenza virus), medications, illicit drugs, alcohol, neuroleptic malignant syndrome, and autoimmune diseases. The mean peak CK reported for each of the various causes and patients with both single and multiple causes ranged from approximately 10,000 to 25,000 IU/L in the largest series [5]; exceptions were three patients with malignant hyperthermia, whose values averaged almost 60,000 IU/L.

Finding the reason for the rhabdomyolysis in our patient was quite puzzling in the beginning. Although we knew that statins could cause muscle damage long after the initiation of the therapy, we still did not find it sufficient to cause such a high elevation of CK. Although the patient did not exhibit any classic symptoms of viral infection, given the current context of the ongoing COVID-19, we still found it necessary to conduct the appropriate tests, which helped to shed light on the etiology of the process.

About a third of the reported virus-induced rhabdomyolysis are caused by influenza [6]. SARS-CoV-2 has been isolated from multiple organs and raises the possibility of the virus infecting striated muscle and potentially leading to muscle breakdown [7]. The mechanism of COVID-induced muscle cell damage remains unknown, but direct viral or toxin-induced injury could be involved similar to other viruses [6]. Another hypothesis is that skeletal muscle damage could be caused by the host “cytokine storm”-like immune response [6]. So far, several case reports linking COVID-19 and rhabdomyolysis have been published, but none of the patients had a CK level of more than 30,000 IU/L, neither were they taking statins. Recent reports showed that rhabdomyolysis could be an initial manifestation of COVID-19, rather than a late complication as previously reported [8].

One of the case series included ten patients with COVID-19 associated with rhabdomyolysis. The median age of the participants was 55 years, and all were male. None of the patients received statins or other medications known to cause rhabdomyolysis or had risk factors for rhabdomyolysis. The median CK level on presentation was 4460 U/L. Three patients had AKI on presentation, and nine patients had transaminitis. Inflammatory markers (erythrocyte sedimentation rate, C-reactive protein, fibrinogen, and ferritin) were elevated in all patients. Other contributing infectious agents (for example parainfluenza, enterovirus, and adenovirus) were not identified, and eight out of ten patients died [9].

Another remarkable aspect is the course and outcomes of rhabdomyolysis in the setting of COVID-19. Since the beginning of the pandemic, it was noted that CK elevation above the upper limit of normal (> 185 U/L) correlated with severe disease and increased mortality [10]. In one study, serum CK decline correlated with viral mRNA elimination [11]. A retrospective observational cohort study conducted in New York included 140 patients with rhabdomyolysis in the setting of COVID-19. The median CK on admission and discharge was 1323 U/L and 852 U/L, respectively. Median creatinine on admission was 1.78 mg/dL. During hospitalization, 49 patients (35%) received invasive mechanical ventilation and 112 patients (80%) developed AKI, 24 (17.1%) of which required renal replacement therapy (RRT). Out of 140 patients, 74 (52.9%) were discharged and 66 (47.1%) died in the hospital. Patients who developed rhabdomyolysis had higher mortality rates and new-onset RRT than patients with COVID-19 with peak CK less than 1000 U/L. However, based on logistic regression models, CK levels on admission and peak CK levels were not statistically significant predictors of either new-onset RRT or mortality in the study group [12].

As for isolated transaminitis, we concluded that, in our case, it stemmed from rhabdomyolysis and COVID-19 hepatotoxicity. In one study, AST was elevated in 93.1% and ALT in 75% of rhabdomyolysis cases in which the CK was greater than or equal to 1000 U/L [13]. In only one instance was the ALT greater than the AST, although the AST declines faster than the ALT as the rhabdomyolysis resolves, such that the two may equalize after a few days [14], which can be seen in our case. An important pathogenesis factor is hepatic inflammation, which is triggered by proteases released from injured muscle [15]. As to COVID-19 itself, the emerging data support the hypothesis that liver injury is often the result of SARS-CoV-2 directly binding to ACE2 positive cholangiocytes, leading to cholangiohepatitis, which a cytokine storm can further exacerbate [16, 17].

The management of rhabdomyolysis consists of aggressive intravenous fluid resuscitation and addressing the causative agent. Correction of electrolyte abnormalities is of utmost importance. Rarely, the patients might need dialysis for persistent acidosis, volume overload, and severe electrolyte abnormalities, but one should keep in mind that dialysis is ineffective in removing myoglobin and uric acid per se. Our patient responded very well to the fluid resuscitation, and his CK, Cr, and liver function test (LFT) levels progressively declined.

In conclusion, we present a case of rhabdomyolysis with highly elevated CK levels as the initial extrapulmonary manifestation of COVID-19. The spectrum of damage of COVID-19 is still an evolving topic, and more research is required to reveal the exact mechanisms by which SARS-CoV-2 leads to rhabdomyolysis; the degree of statin contribution to this process should be studied as well. It is yet to be elucidated if rhabdomyolysis serves as a predictor of poor outcome and mortality in COVID-19. Clinicians should be cognizant of this rare and atypical initial presentation of COVID-19.

## Data Availability

The data used to support the findings of this study are available from the corresponding author on request, except for the patient’s personal health information due to Health Insurance Portability and Accountability regulations.

## Abbreviations

ACE2: Angiotensin-converting enzyme 2
CK: Creatine kinase
Cr: Creatinine
COVID-19: Coronavirus disease 2019
AKI: Acute kidney injury
ALT: Alanine aminotransferase
AST: Aspartate aminotransferase
GGT: Gamma-glutamyl transferase
HIV: Human immunodeficiency virus
HST: High-sensitivity troponin
LFT: Liver function test
mRNA: Messenger ribonucleic acid
PCR: Polymerase chain reaction
RRT: Renal replacement therapy
SARS-CoV-2: Severe acute respiratory syndrome coronavirus 2
